# Draft Genome Sequences of Four Strains of Campylobacter jejuni Isolated from Patients with Axonal Variant of Guillain-Barré Syndrome in Bangladesh

**DOI:** 10.1128/mra.01146-21

**Published:** 2022-02-10

**Authors:** Shoma Hayat, Fahmida Habib Nabila, Asaduzzaman Asad, Ruma Begum, Israt Jahan, Hubert P. Endtz, Zhahirul Islam

**Affiliations:** a Laboratory of Gut-Brain Signaling, Laboratory Sciences and Services Division (LSSD), International Centre for Diarrhoeal Disease Research (icddr,b), Dhaka, Bangladesh; b Department of Medical Microbiology and Infectious Diseases, Erasmus MC, University Medical Centre Rotterdam, Rotterdam, The Netherlands; Loyola University Chicago

## Abstract

Four Campylobacter jejuni strains (Z191005RS, Z191005SS, Z201020RS, and Z201020SS) isolated from the axonal variant of Guillain-Barré syndrome (GBS) were sequenced using Illumina technology. The average genome size was from 1.61 to 1.63 gb, with a very high coverage ranging from 654× to 758×, which facilitates the possibility of rare variant calling.

## ANNOUNCEMENT

Campylobacter jejuni is the predominant preceding infectious agent in the axonal variant of Guillain-Barré syndrome (GBS) (1). Certain C. jejuni strains have a ganglioside-like structure in lipo-oligosaccharides (LOSs) and induce molecular mimicry-driven GBS (1–3). The detailed mechanism of this cross-reactive autoimmunity is still unknown (1, 4). Bacterial genetics are entailed to be assessed in evaluating the virulence factors responsible for inducing axonal GBS (4). Periodical genome profiling of C. jejuni strains isolated from a patient with an axonal variant of GBS would provide features revealing their detailed versatile pathogenesis mechanism. It also helps in comparing the mechanisms among different C. jejuni strains causing different types of campylobacteriosis and variation in subtypes of GBS. The study was reviewed and approved under protocol number PR-19048 by the institutional review board (IRB) of icddr,b, Bangladesh.

Four C. jejuni strains were isolated from rectal swabs or stool specimens (Z191005RS and Z191005SS from one and Z201020RS and Z201020SS from another patient) of two patients with acute motor axonal neuropathy (AMAN), using standard microbiological procedures (4). The C. jejuni isolates were selected and enriched for 48 h at 42°C in Brucella agar and blood agar with 5% sheep blood, respectively. Selected C. jejuni isolates were confirmed by species-specific PCR. Genomic DNA was extracted using the Wizard genomic DNA purification kit (Promega) followed by quality and quantity check using a NanoDrop spectrophotometer (Thermo Scientific) and Qubit 2.0 fluorimeter (Life Technologies), respectively (5). The sequencing library was constructed using 1 ng of genomic DNA employing Illumina Nextera XT DNA library preparation kit. Sequencing was performed using the Illumina NextSeq 500 platform employing the Illumina NextSeq v2.5 reagent kit (2,150 bp) (Illumina platform) (6). Quality checks on the paired-end sequencing reads (150 bp) were performed using FastQC v0.11.9 (7). The genome coverage was found to be 654× to 758× by mapping the reads against the reference genome Campylobacter jejuni ATCC 700819 (GenBank accession number NC_002163.1) using BWA v0.7.17-r1188 and SAMtools v1.14 (8, 9). Trimmomatic v0.36 and fastp v0. 19.5 were used for adapter trimming and quality trimming, respectively (10, 11). *De novo* assembly was performed using SPAdes v3.14.1 adding “--isolate” flag and QUAST v5.0.2 to assess assembly statistics (12, 13). NCBI Prokaryotic Genome Annotation Pipeline (PGAP) v5.1 was used to annotate the assembled draft genomes (14). Clustered regularly interspaced short palindromic repeats (CRISPR) arrays were interpreted and analyzed on the CRISPRCasFinder and the CRISPRTarget web tools (15, 16). Default parameters were applied for all software unless otherwise specified.

All four sequences have a coverage >650 with a G+C content around 30%. PGAP annotated a total of 1,692 to 1,720 genes, including 45 to 48 RNAs in the C. jejuni strains. Moreover, Z191005RS and Z191005SS strains had similar genome sequences (99.99%) calculated by the average nucleotide identity (ANI) calculator (17). Both genomes possessed the same CRISPR arrays and spacer sequences. The protospacer from Campylobacter phage DA10 (GenBank accession number MN530981) was commonly targeted by both strains, which is similar to the previously announced C. jejuni strains (18). Finally, these draft genome sequences would help to determine strain-level differences and identify genetic variants/genes associated with axonal GBS pathogenesis.

### Data availability.

All mentioned draft genomes were deposited in GenBank under BioProject accession no. PRJNA717137 (Table 1).

**TABLE 1:** Genome data and accession numbers of strains of Campylobacter jejuni Z191005RS, Z191005SS, Z201020RS, and Z201020SS

Strain	Source	Genome size (bp)^*a*^	No. of reads	G+C content (mol %)	No. of contigs (*N*_50_ [bp]^*a*^)	GenBank accession no.	Total no. of genes	No. of rRNAs (5S, 16S, 23S)	No. of tRNAs	SRA^*b*^ accession no	Genome coverage (×)
Z191005RS	Rectal swab	1,628,993	3,897,758	30.57	55 (153,957)	JAGIQH000000000	1,718	1, 1, 1	40	SRR14206027	682
Z191005SS	Stool	1,631,208	4,319,713	30.62	55 (153,957)	JAGIQI000000000	1,720	1, 1, 1	40	SRR14205973	758
Z201020RS	Rectal swab	1,622,648	4,526,623	30.64	60 (183,722)	JAGIQJ000000000	1,714	1, 2, 1	41	SRR14206028	730
Z201020SS	Stool	1,616,053	4,057,623	30.55	35 (183,722)	JAGIQK010000000	1,692	1, 1, 1	39	SRR14205974	654

a bp, base pair.

b SRA, Sequence Read Archive.
